# SinEx DB: a database for single exon coding sequences in mammalian genomes

**DOI:** 10.1093/database/baw095

**Published:** 2016-06-07

**Authors:** Roddy Jorquera, Rodrigo Ortiz, F. Ossandon, Juan Pablo Cárdenas, Rene Sepúlveda, Carolina González, David S. Holmes

**Affiliations:** Center for Bioinformatics and Genome Biology, Fundacion Ciencia & Vida and Facultad de Ciencias Biologicas, Universidad Andres Bello, Avda Zañartu 1482, Santiago, Chile

## Abstract

Eukaryotic genes are typically interrupted by intragenic, noncoding sequences termed introns. However, some genes lack introns in their coding sequence (CDS) and are generally known as ‘single exon genes’ (SEGs). In this work, a SEG is defined as a nuclear, protein-coding gene that lacks introns in its CDS. Whereas, many public databases of Eukaryotic multi-exon genes are available, there are only two specialized databases for SEGs. The present work addresses the need for a more extensive and diverse database by creating SinEx DB, a publicly available, searchable database of predicted SEGs from 10 completely sequenced mammalian genomes including human. SinEx DB houses the DNA and protein sequence information of these SEGs and includes their functional predictions (KOG) and the relative distribution of these functions within species. The information is stored in a relational database built with My SQL Server 5.1.33 and the complete dataset of SEG sequences and their functional predictions are available for downloading. SinEx DB can be interrogated by: (i) a browsable phylogenetic schema, (ii) carrying out BLAST searches to the in-house SinEx DB of SEGs and (iii) via an advanced search mode in which the database can be searched by key words and any combination of searches by species and predicted functions. SinEx DB provides a rich source of information for advancing our understanding of the evolution and function of SEGs.

**Database URL**: www.sinex.cl

## Introduction

In most Eukaryotic genes, the coding sequence (CDS) is interrupted by noncoding introns that are removed by splicing to generate mRNA. However, some single exon coding genes, also termed single exon genes (SEGs), have been identified. Although some SEGs are thought to be processed pseudo-genes, many have been demonstrated to be expressed (1–3), raising questions as to their origin, evolution and function.

Among the well characterized SEGs, with experimentally verified functions, are multiple genes encoding histones (4), G protein coupled receptors (GPCRs) (5–7), olfactory receptors (8), transcription factors and proteins involved in the regulation of development, growth and proliferation (9). There is evidence that SEGs tend to be expressed in a tissue specific manner. For example, it has been proposed that the expression of a large proportion of human SEGs is testis and neuro-specific and associated with several types of cancer, neurological and developmental disorders (9).

Investigations into the origin and evolution of SEGs within the Eukaryote domain suggest that many SEGs have arisen from multi-exon genes via retrotransposition (8, 10) and databases of predicted retrotransposed genes, including SEGs, have been constructed (11, 12). Other molecular mechanisms for the origin of SEGs have been proposed such as de novo origin (13), DNA-based duplication from intron-containing genes (14), and intron loss, among others (15, 16). It has also been suggested that SEGs evolve significantly faster than intron-containing genes (17).

Whereas a large number of searchable databases for intron containing genes are available, e.g. (18, 19), there are only two publicly available databases for SEGs. These are IGD, housing a collection of human intronless genes (20) and PIGD, a SEGs database from five plants (21). A database of SEGs from five Eukaryotic genomes published a decade ago is no longer available (22). Herein, we extend and complement the IGD and PIGD databases by creating SinEx DB, a publicly available searchable database of predicted SEGs from 10 completely sequenced mammalian genomes, namely: human, chimpanzee, rhesus macaque, mouse, rat, dog, horse, pig, cow and opossum. SEGs deposited in the SinEx DB include examples that are predicted to have arisen by mechanisms other than retrotransposition and, therefore, SinEx DB extends the information available in Retrogene DB (11).

In SinEx DB, a SEG is defined as a nuclear (nonmitochondrial), protein-coding gene that lacks introns in its CDS. The definition excludes genes that generate functional RNAs such as tRNA, rRNA and regulatory RNAs. It also excludes potential genes that do not contain the ‘CDS FEATURE’ convention in their annotation. An additional consideration is that SEGs could still contain introns in their 5′ and/or 3′-untranslated region (UTR). SEGs, annotated as ‘pseudogenes’ were binned separately and were not included in this first version of SinEx DB.

It is anticipated that SinEx DB will prove a useful resource for addressing questions regarding the occurrence, genomic and functional distribution of SEGs on a large comparative genome scale.

## Database construction

The sequences of annotated mammalian genomes, assembled at a chromosome level, were downloaded from GenBank (23) at the FTP site on the NCBI web page (ftp://ftp.ncbi.nlm.nih.gov/genomes/), including: human (ref_GRCh37.p5), chimpanzee (ref_Pan_troglodytes-2.1.4), rhesus macaque (ref_Mmul_051212), mouse (ref_MGSCv37), rat (ref_RGSC_v3.4), dog (ref_CanFam2.0), horse (ref_EquCab2.0), pig (ref_Sscrofa10), cow (ref_Btau_4.2) and opossum (ref_MonDom5). Using Perl scripts and BioPerl Application Programming Interface (API) (24), CDS gene identifiers in the Genbank-format chromosome files corresponding to amino acid CDSs, were selected according to their location coordinates and classified into two groups, namely: those that contained only 1 pair of start and end, e.g. ‘1.30’ (SEGs) and those that contain multiple pairs of start and end coordinates, e.g. ‘join(1.30,50.109 121.150)’ (multi-exon genes or MEGs). MEGs were binned and stored separately and can be downloaded from SinEx DB. Additionally, all CDS containing the ‘/pseudo’ tag (annotated as inactive pseudogenes) were binned and stored separately (Supplementary Table S1). Genes from mitochondrial genomes were not included in the database generation.

**Figure 1.**
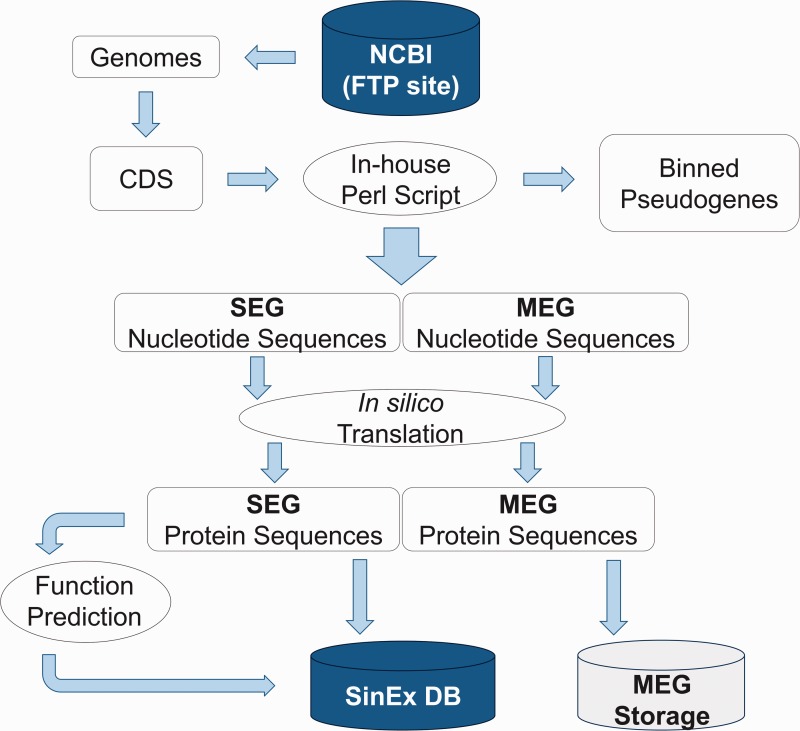
Bioinformatic pipeline outlining the strategy for SinEx DB construction. Ten sequenced mammalian genomes (see text for list) were downloaded from the FTP site in the NCBI web page (ftp://ftp.ncbi.nlm.nih.gov/genomes/). Nucleotide sequences were translated *in silico* to corresponding amino acids. Using Perl scripts and BioPerl Application Programming Interface (API) genes were parsed into single exon (SEGs) and multi-exon genes (MEGs). MEGs and annotated pseudogenes were binned and stored separately.

High dimensional analysis of the SEG information includes their distribution within genomes and prediction of their function. Sequences were categorized using KOG (25) and RPS-BLAST (26), implemented in an in-house platform developed in Perl. The information was stored in a relational database built with My SQL Server 5.1.33 (Figure 1).

The quality of the genome sequence and annotation will influence the prediction of single-exon coding sequences. In order to minimize this problem, SinEx DB is constructed using only completely sequenced and annotated mammalian genomes, assembled at a chromosome level.

## Data content

SinEx DB provides information regarding the occurrence, properties and genomic distribution of 31 624 SEGs out of a total of 248 152 annotated CDSs from 10 completely sequenced mammalian genomes.

SEGs annotated as pseudogenes in the NCBI database were binned separately (Supplementary Table S1) and are not included in SinEx DB. Thus, SinEx DB complements the database of eukaryotic pseudogenes available at pseudogene.org (27).

The average percentage of SEGs to total protein encoding genes within the 10 mammalian genomes is 12.9%, with no statistically significant difference between genomes (SD = ±3.05). The occurrence of SEGs ranges from 8.9% in human (*Homo sapiens*) to 17.3% in rat (*Rattus norvegicus)* (Table 1). The percentage of predicted SEGs in the human genome is lower than the 12.3% previously reported in 2004 (28), probably due to recent improvements in the annotation of the human genome.

**Table 1.** **** Occurrence of total annotated CDS by NCBI, gene density [gene/genome size (Mb)] and predicted single exon genes in mammals using in-house Perl script.

Species	Name	Total CDS	SEG number	^Δ^SEG percentage	Av. SEG length^a^	Genome size (Mb)^b^	Gene number^b^	Gene density (gene/Mb)	Total gene number with 5′ and/or 3′-UTRs^c^
*Homo sapiens*	Human	35 195	3128	8.9	341	2670.42	27 155	10.2	21 838
*Pan troglodytes*	Chimpanzee	33 726	3522	10.4	306	2528.45	24 440	9.7	20 583
*Macaca mulatta*	Macaque	29 288	4713	16.1	220	1412.47	28 770	20.4	17 376
*Mus musculus*	Mouse	28 789	4858	16.9	302	2474.93	22 900	9.3	25 553
*Rattus norvegicus*	Rat	19 402	3355	17.3	297	3095.69	37 150	12.0	18 679
*Canis lupus familiaris*	Dog	21 894	2392	10.9	305	3600.5	21 583	6.0	1161
*Equus caballus*	Horse	20 210	1953	9.7	328	3097.59	29 413	9.5	7567
*Sus scrofa*	Pig	22 663	2703	11.9	302	2654.91	34 293	12.9	5277
*Bos taurus*	Cow	18 577	2551	13.7	286	3160.37	30 235	9.6	18 039
*Monodelphis domestica*	Opossum	18 410	2449	13.3	330	2725.99	29 100	10.7	1329

Percentage of predicted SEGs (Δ) as a function of total annotated CDS per genome.

a Average CDS length of SEGs in amino acids.

b Obtained from NCBI web page (http://www.ncbi.nlm.nih.gov/genome/).

^c^Obtained from UTRdb web page (http://utrdb.ba.itb.cnr.it/home/statistics).

The average potential coding capacity of the SEGs is 302 amino acids and ranges from 220 in Rhesus macaque (*Macaca mulatta*) to 341 in human (*Homo sapiens*), with no statistically significant difference between genomes (SD = ±33) (Table 1).

There is no statistical correlation between the number of total genes with 5′- and/or 3′-untranslated regions (UTRs) (29) and SEG percentage (R2 = 0.5009) among the analyzed mammalian genomes (Supplementary Figure S1).

## Web interface

**Figure 2.**
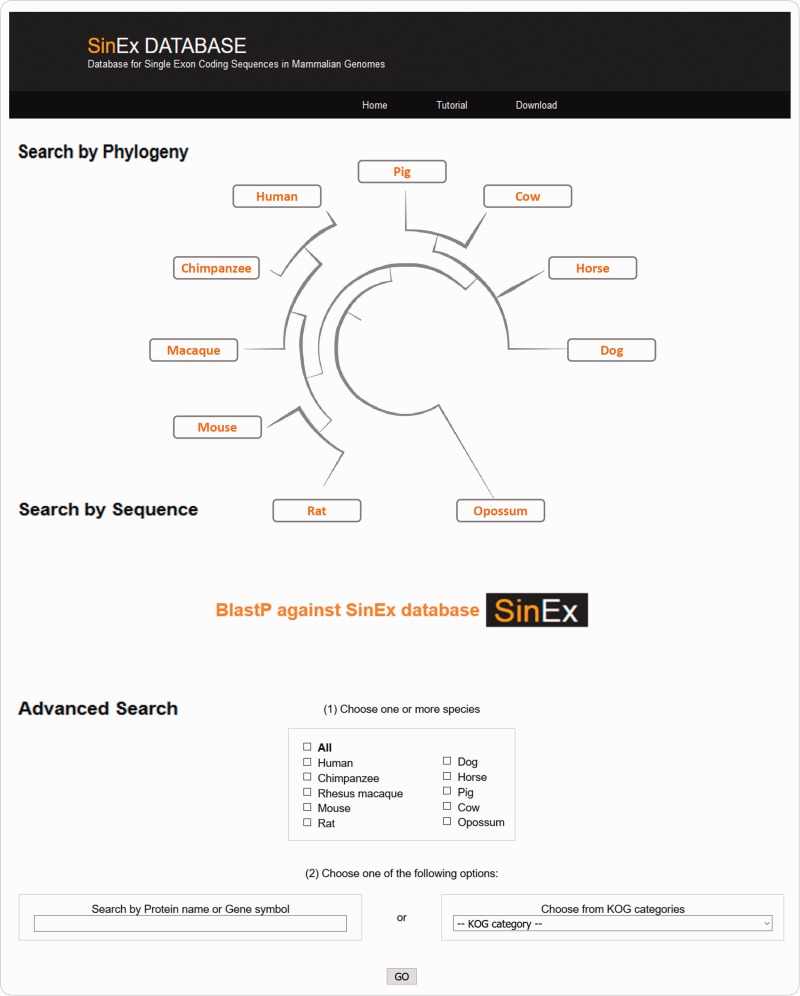
Screen shot of the web interface of SinEx DB. There are three ways to access SinEx DB data: (i) by exploring the database content through the browsable phylogenetic schema, (ii) using a protein sequence in FASTA format as a query against SinEx DB and (iii) doing an advanced search to interrogate one or more genomes (see text for more details). Nucleotide and protein sequences of SEGs and protein sequences of MEGs from 10 mammalian genomes are downloadable in FASTA format. A tutorial is also available in the webpage (www.sinex.cl/tutorial.app).

There are three ways to access SinEx DB data via the web interface (Figure 2): (i) through the browsable phylogenetic schema, in which the user can obtain KOG functional categories for SEGs, can access their predicted protein sequences and perform external searches, (ii) by interrogating a protein sequence as a query in BLASTP against the in-house SinEx DB and (iii) by performing an advanced search using ‘genome’, ‘protein name’, ‘protein ID’, ‘chromosome number’, ‘gene symbol’, or by ‘KOG category’ or combinations of the above to search the in-house SinEx DB. The search by protein name is not case sensitive but is sensitive to different spelling. Hot-links to NCBI sequence accession entries were included for all sequences within the SinEx DB web interface.

Nucleotide and protein sequences of SEGs and protein sequences of MEGs from 10 mammalian genomes included in SinEx DB are downloadable in FASTA format. A section of statistical information of occurrence and functional classification of SEGs in mammalian genomes and a tutorial to facilitate user’s recovery of data are also available in the webpage (www.sinex.cl/tutorial.app).

## Utility

SinEx DB can be used for investigating the function of SEGs, both within a specific genome and across multiple genomes and can facilitate other aspects of comparative genomics. It could also be useful for generating models of SEGs evolution, including the timing of appearance or disappearance of SEGs across the phylogenetic tree.

Example of the use of SinEx DB: comparative analysis of SEG functions in mammals.

**Figure 3.**
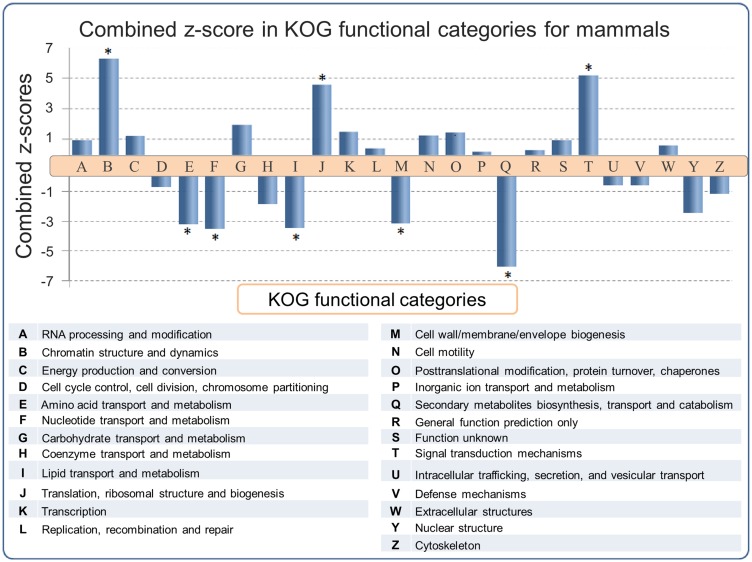
SEG/MEG proportion in different KOG functional categories for mammals, represented as a combined *z*-score from multiple tests (mammals). A high dimensional analysis of all categorized sequences from SinEx DB shows that, in mammals, CDSs with predicted functions related to chromatin structure (B), signal transduction mechanisms (T) and translation (J) are enriched in SEGs (high proportion of SEGs to MEGs), whereas CDSs with predicted functions related to envelope biogenesis, amino acid, nucleotide, secondary metabolites and lipid metabolism have the lowest SEGs to MEGs proportion. The *P* value was obtained using the Pearson’s chi-squared test and corrected by Sidak multiple testing method (31). Asterisk indicates statistical significance, *P* < 0.05.

Functional predictions of SEGs have been reported for the human (6, 8, 20, 30) and mouse (2) genomes. However a large scale multiple-genome comparison of predicted SEG functions has not been carried out. In order to address this issue, SEGs and MEGs derived from the SinEx DB were binned into 25 functional categories using the KOG classifier (25). Subsequently, the ratio of SEGs to MEGs in each functional category of each genome was calculated in order to determine if any KOG functional category was enriched in SEGs. To evaluate the statistical significance of the results, the natural logarithm of the ratio of SEGs to MEGs [Ln (SEG/MEG)] was obtained and the average and standard deviation of Ln (SEG/MEG) within each species were used to calculate *Z*-scores and to normalize data (Figure 3). *P* values using the Pearson’s chi-squared test was calculated and corrected with the Sidak method for multiple comparisons (31). The higher the *z*-score, the more distant it is from the population mean.

The results indicate that the distribution of SEGs within the functional categories of the different genomes is nonuniform, with statistical support (*P* < 0.05) in some instances. For example, an enrichment of SEGs relative to MEGs was observed in functions related to: (i) chromatin structure and dynamics including histones, (ii) signal transduction mechanisms including G protein-coupled cell surface receptors (GPCRs) and (iii) translation related proteins, such as ribosomal proteins (Figure 3). These observations support and extend the earlier reports for the distribution of SEGs in human (6, 8, 20, 30) and mouse (2). The enrichment of SEGs for histones has been proposed to expedite their rapid synthesis and high levels of gene expression during DNA replication (6). Consistent with this hypothesis, is the observation that introns are found in replacement histone genes which have a cell-cycle-independent pattern of expression (32). It has been established earlier that GPCRs, one of the largest classes of mammalian receptor, are predominantly intronless (5–7). The majority of GPCR genes are related to nervous central system (CNS) activities, which often require high levels of gene expression.

There is also an enrichment of SEGs relative to MEGs potentially encoding ‘translation related functions’ (KOG category J) in all the mammalian genomes analyzed (Figure 3). 83% (1499 sequences) of these SEGs potentially encode ribosomal proteins. The reason for this enrichment is unknown and calls for an explication(s). Many of these ribosomal proteins are highly expressed in a cell cycle-dependent way (33) and they could be encoded by SEGs for reasons similar to those proposed for histone genes. However, some of the ribosomal proteins encoded by SEGs exhibit diverse functional roles such as in ribosome biogenesis, cell proliferation, differentiation, apoptosis and DNA repair (34, 35) and these functions might also play a role in SEG enrichment.

On the other hand, there is a depletion of SEGs relative to MEGs in genes with predicted functions related to: (i) amino acid transport and metabolism, (ii) nucleotide transport and metabolism, (iii) lipid transport and metabolism, (iv) cell wall/membrane/envelope biogenesis and (v) secondary metabolites biosynthesis, transport and catabolism. These categories are important components of central metabolism and part of the core genome of mammals (36, 37). We hypothesize that these categories are depleted in SEGs (i.e. enriched in introns) because introns may play an important role in increasing genetic diversity through alternative splicing. It has also been suggested that introns may carry out regulatory functions to coordinate expression of genes that are components of metabolic networks, such as those found in the above five categories, and thus be under positive selection for introns (6, 38, 39).

## Conclusions

SinEx DB provides an opportunity to address questions regarding the occurrence, distribution, evolution and function of single exon coding sequences (SEGs) in 10 diverse mammalian genomes. SinEx DB complements existing databases such as retrogene DB (11) and pseudogene DB (27). It could also be used as a comparative platform for annotating single exon coding sequences in mammalian genomes.

Interrogation of SinEx DB was used successfully to correlate the enrichment or depletion of SEGs with predicted KOG functional categories, uncovering new relationships between SEGs and gene function that now require explanation.

## Future perspectives

It is proposed to update SinEx DB twice a year with annotated SEGs from additional completely sequenced eukaryotic genomes, ranging from unicellular eukaryotes to other mammals. Future versions of the database will include alternative functional GO classifications, this may complement KOG classifications and allow more detailed functional analysis, information related to predicted introns in the UTR regions of SEGs. This could be an important resource considering the relevance of UTR introns in cellular biology and gene expression (40–42).

We propose that SEGs from different and diverse genomes available in future versions of SinEx DB will provide additional opportunities to analyze many-to-many comparisons between genomes.

## Availability and requirements

SinEx DB is freely and publicly available at http://www.sinex.cl and the complete dataset is available for download by ftp.

## Supplementary data

Supplementary data are available at *Database* Online.

Supplementary Data
