# Supplementary material for: SinEx DB: a database for single exon coding sequences in mammalian genomes
Source: Database (Oxford). 2016 Jun 7;2016:baw095. doi: 10.1093/database/baw095 (PMC4897596; doi:10.1093/database/baw095)
Supplement: Supplementary Data [file supp_2016_baw095_index.html]

Supplementary Data 

# SinEx DB: a database for single exon coding sequences in mammalian genomes

## Supplementary Data

files

- Supplementary Data - docx file
- Supplementary Data - docx file
