# Supplementary material for: SinEx DB: a database for single exon coding sequences in mammalian genomes
Source: Database (Oxford). 2016 Jun 7;2016:baw095. doi: 10.1093/database/baw095 (PMC4897596; doi:10.1093/database/baw095)
Supplement: Supplementary Data [file supp_baw095_Supplementary_Figure_S1.docx]

Supplementary material

**Supplementary Figure S1:**


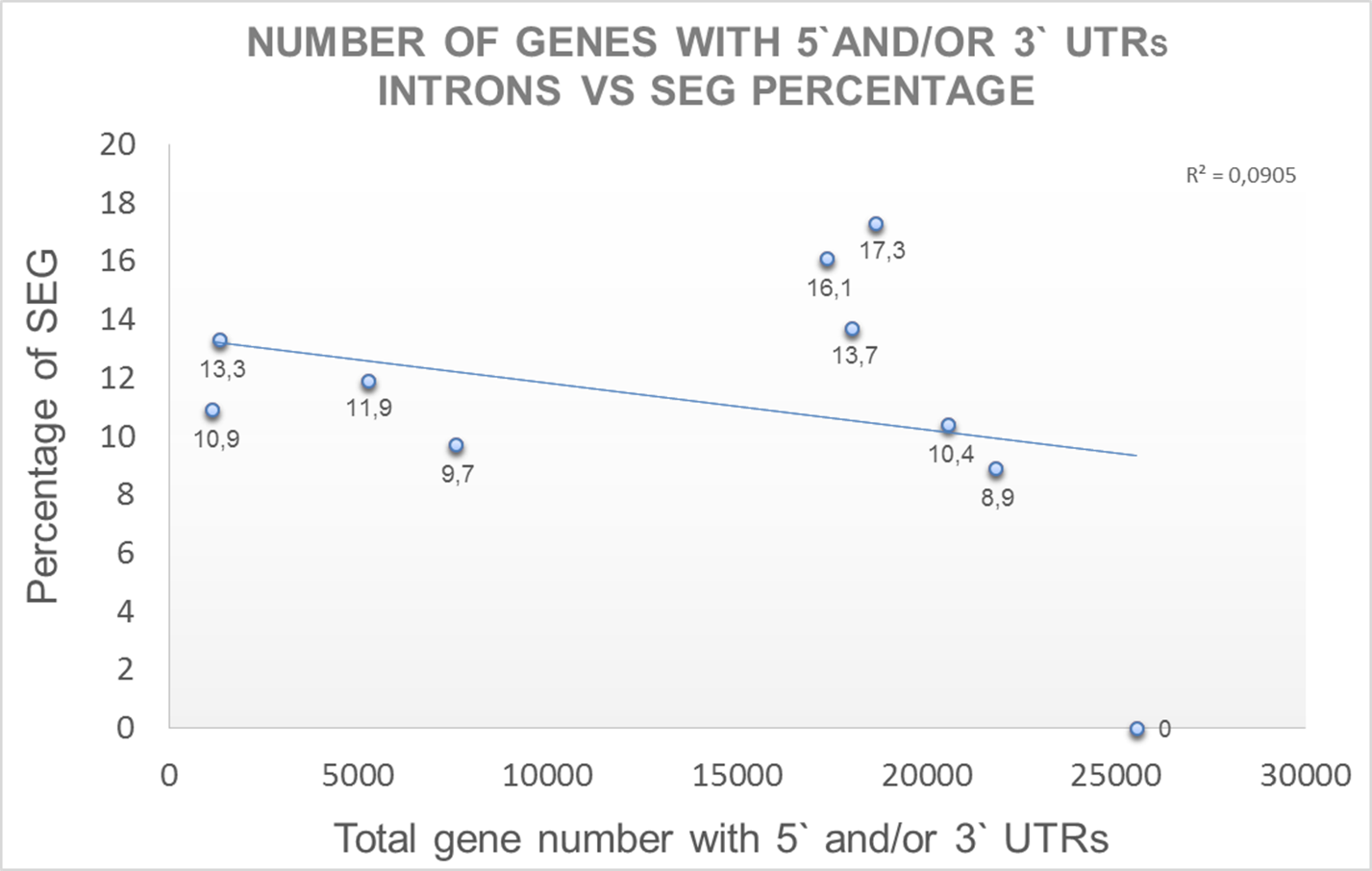


**Supplementary Figure S1**: Correlation between number of genes with 5' and/or 3' untranslated region (UTR) and SEG percentage in 10 mammalian genomes included in SinEx DB.
