# Supplementary material for: SinEx DB: a database for single exon coding sequences in mammalian genomes
Source: Database (Oxford). 2016 Jun 7;2016:baw095. doi: 10.1093/database/baw095 (PMC4897596; doi:10.1093/database/baw095)
Supplement: Supplementary Data [file supp_baw095_Supplementary_Table_S1.docx]

Supplementary material

**Supplementary Table S1:**


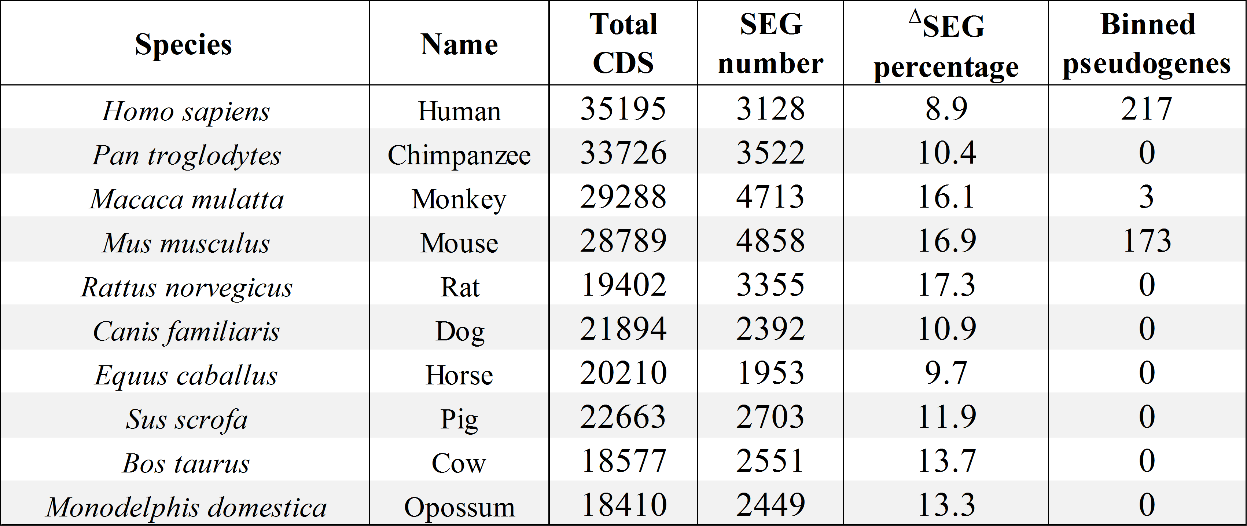


**Supplementary Table S1:** Occurrence of total annotated CDS by NCBI, predicted single exon genes in mammals using in-house Perl script and number of annotated pseudogenes binned. Percentage of predicted SEGs (Δ) as a function of total annotated CDS per genome.
